# A preconception intervention targeted at women with modifiable risk factors before pregnancy to improve outcomes; protocol for the Get Ready! feasibility trial

**DOI:** 10.1186/s40814-021-00824-0

**Published:** 2021-03-26

**Authors:** Angela C. Flynn, Emma Pryke, Monal Wadhera, Lucilla Poston, Sara L. White

**Affiliations:** 1 Department of Women and Children’s Health, School of Life Course Sciences, King’s College London, London, UK; 2 LiveSmart, 37 Cremer Street, London, UK

**Keywords:** Preconception, Health behaviours, Diet, Physical activity, Metabolic health, Pregnancy complications

## Abstract

**Background:**

The health of a woman before conception not only influences the outcome of her pregnancy but also the lifelong health of mother and child. Many women in the UK are inadequately prepared for pregnancy, with reports of a high prevalence of smoking, low folic acid supplement use, and suboptimal diet and physical activity. Get Ready! will link an online digital tool to identify women planning pregnancy most at risk of complications with a personalised intervention to improve health behaviours and biomarkers of metabolic health.

**Methods:**

Women planning pregnancy will be identified from a free and widely used online preconception tool. A short online screening questionnaire will then be used to recruit women considered to be at high metabolic risk. Eligibility criteria include resident in the UK, age > 18–< 50 years, BMI ≥ 23 kg/m^2^ (South Asian) or ≥ 25 kg/m^2^ (all other ethnicities), and plus one or more of the following: 1st degree relative with type 2 diabetes, previous gestational diabetes (GDM), previous baby > 4 kg, or high risk ethnicity for GDM. Eligible women who consent to participate will be enrolled in a commercially available preconception intervention (Prepare Plans, LiveSmart UK Ltd). Following an online health assessment and home blood test, women will be provided with individualised lifestyle advice and coaching by dietitians. Process evaluation will provide an assessment of implementation of the intervention. Change in health behaviours and biomarkers of metabolic health will also be examined.

**Discussion:**

Suboptimal health behaviours amongst women planning pregnancy are widely prevalent in the UK. Personalised health checks and coaching are especially important for women at risk of pregnancy complications. Get Ready! introduces a novel approach to identifying high risk women planning pregnancy and provision of a targeted intervention.

**Registration:**

Trial sponsor: King’s College London.

## Background

Health behaviours before pregnancy may profoundly influence pregnancy outcomes, and the future health of both mother and child, as detailed in a recent Lancet series [1]. The increasing awareness of health in the preconception period has been precipitated, at least in part, by the high prevalence of obesity amongst women at their first antenatal visit. Over half of women in the United Kingdom (UK) were overweight or obese in 2016–2017 [2], which increases the risk of adverse maternal, infant, and childhood outcomes and is a major burden to National Health Service (NHS) antenatal services and cost resources [3, 4]. This, together with the greater recognition that lifestyle changes are better made before, rather than during pregnancy has stimulated a shift in focus to strategies which promote healthier lifestyles amongst women contemplating pregnancy [1]. Aside from adverse influences on maternal body mass index, suboptimal nutrition per se is a common and modifiable cause of concern, as micronutrient deficiencies are implicated in fetal growth and development [5]. Data from the UK suggests that women of reproductive age are not nutritionally prepared for pregnancy, particularly younger women since they do not meet recommendations for micronutrient intakes including iron, iodine, and folate [1]. The benefit of folic acid supplementation in prevention of neural tube defects is indisputable [6], but in a recent survey of over 130,000 UK women planning pregnancy, we have shown that less than one third took folic acid supplements (Sara L. White, personal communication). Similarly, although the detrimental effects of smoking in pregnancy are widely known [7], 20% of women reported smoking in our survey (Sara L. White, personal communication).

Achieving readiness for pregnancy through appropriate preconception planning is key to optimising pregnancy outcomes for all women, with important consequences for maternal and child health. Surveys undertaken using the London Measure of Unplanned Pregnancy (LMUP) questionnaire suggest that pregnancy planning is common [8–10], although, currently, few NHS services target women planning pregnancy, with the majority of resources focusing on the antenatal period. NHS preconception care in the UK broadly falls within the remit of primary care and sexual health services [11]. Women planning pregnancy are often a difficult to reach group and are more likely to engage with health services once they become pregnant rather than before. Moreover, preconception care has been reported by primary care providers as being constrained by time and resources as well as competing with other preventative care [12]. Therefore, the first barrier to intervention is identifying women planning pregnancy.

Trials of interventions to improve health before conception are few [13] and use an unselected approach in targeting the general preconception population. Targeted personalised intervention in higher risk women planning pregnancy may be a more efficient and beneficial approach. Through an online recruitment process, this study will evaluate a novel approach to both identification of women planning pregnancy and those most at risk and examine whether a targeted intervention will improve health behaviours and markers of metabolic health.

## Methods

This protocol paper has been written following the SPIRIT guidance [14].

### Study aim

The aim of this study is to assess the feasibility of using an online recruitment process to identify high risk women planning pregnancy and examine health behaviours and biomarkers of metabolic health after a personalised intervention.

### Study objectives

- To assess feasibility of linking users of a freely accessible online preconception tool via an online consenting process to an established preconception intervention.
- To estimate the rate of participation of women who are eligible to be enrolled into the study.
- To deliver a preconception intervention, informed by evidence-based lifestyle advice.
- To measure change in health behaviours including diet, physical activity, micronutrient supplementation, smoking, alcohol, and biomarkers of metabolic health.
- To perform a process evaluation.
- To use the data to determine participant numbers needed, intervention strategies, and methods of data collection and inform a primary outcome for a randomised controlled trial.

### Study design

**Fig. 1:**
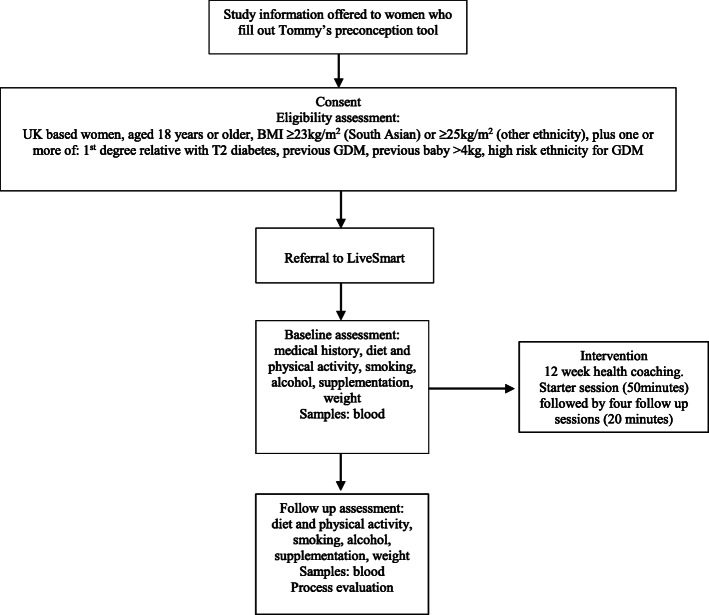
Study flow diagram

This is a single-arm feasibility trial. A flow diagram is shown in Fig. 1.

### Study population

The target population will be women planning a pregnancy considered to be at high risk as defined below.

### Study sample size

The sample size for the feasibility study is based on our objective to estimate the rate of participation of eligible women into the study. This study will recruit 55 women planning pregnancy. If we identify 110 eligible subjects, we will be able to estimate a participation rate of 50% to within a 95% confidence interval of ± 10% [15].

### Inclusion criteria

Women will be eligible for recruitment if they meet the following criteria:

- Age > 18–< 50 years
- Resident in the UK
- Body mass index (BMI) ≥ 23 kg/m^2^ (South Asian) or ≥ 25 kg/m^2^ (other ethnicities)

Plus one or more of the following:

- 1st degree relative with type 2 diabetes
- Previous gestational diabetes mellitus (GDM)
- Previous baby > 4 kg
- High risk ethnicity for GDM including women from South Asian, Black Caribbean, and Middle Eastern ethnicities [16].

### Exclusion criteria

These include:

- Pregnancy
- Resident outside the UK
- Low risk by the above criteria

### Participant recruitment

Women planning pregnancy will be identified from a free and widely used online preconception tool developed by Tommy’s, UK (www.tommys.org/pregnancy-information/planning-pregnancy/planning-for-pregnancy-tool). Users of the tool who leave their email address will receive an automated email inviting participation in the study via a link to the Get Ready! webpage. This webpage allows access to the study information leaflet, consent form, and a short screening questionnaire to assess eligibility. Women interested in participating, who meet the eligibility criteria, and who provide consent will be electronically enrolled to take part in an intervention to improve preconception health.

### Preconception intervention

Women will be directed to receive LiveSmart ‘Prepare Plans’, developed by NHS medical practitioners specifically for women planning a pregnancy (www.getlivesmart.com). The Plans combine online health assessment, blood tests, and personalised health coaching as appropriate for each woman, to optimise diet, preconception micronutrient supplementation, physical activity, and weight management and provide advice on smoking and alcohol reduction as necessary.

### Baseline assessment

The baseline assessment will consist of an online health questionnaire and blood profiling. The questionnaire will include social and demographic data (age, ethnicity, educational attainment, living environment), self-reported data on dietary intake, physical activity, alcohol consumption, smoking habits, anthropometry including weight and waist circumference, and any relevant medical history. For the blood sample, participants will be sent a home blood test kit to obtain a fasting blood sample (2 tubes of 0.5 ml and 0.8 ml) using a simple finger prick device with instructions. The blood sample will be posted to the diagnostic laboratory (County Pathology; http://www.countypathology.co.uk/) for assessment of lipid profile, HbA1c, thyroid function tests, renal function, liver function, and nutritional biomarkers. If abnormal results, e.g. raised HbA1c, are detected, women will be directed into clinical services. The data for the baseline assessment will be analysed by LiveSmart doctors and dietitians to devise individualised recommendations on preconception health improvement. The participants receive a personal health report.

### Health coaching

Individualised health coaching, delivered by dietitians by telephone over 12 weeks, will target modifiable preconception risk factors and provide ongoing support to improve health. The health coaching will include 1 starter session (50 min), plus 4 follow-up sessions (20 min). Throughout the coaching, each participant will be encouraged to identify areas which would benefit from change and as such is highly personalised.

The health coaching will focus on:

- Achieving sustainable weight reduction via lifestyle changes in dietary intake and physical activity.
- Improving dietary intake and physical activity in line with UK national recommendations.
- Appropriate supplementation for women planning pregnancy. Participants will be encouraged to take folic acid (400 μg) and vitamin D (10 μg) supplements in line with national guidelines. Participants will have the option of LiveSmart providing supplements free of charge (Vitl; https://vitl.com). If a woman has a BMI ≥ 30 kg/m^2^, they will be directed to their GP to obtain folic acid 5 mg as per clinical guidance. If a woman has a detected vitamin D deficiency or insufficiency, they will be advised to take a higher dose of vitamin D by the LiveSmart doctor.
- Smoking reduction if appropriate.
- Alcohol reduction if appropriate.

Participants will be encouraged to set achievable goals which will be reviewed in follow-up sessions. Participants will also be encouraged to identify potential barriers to implementation of their lifestyle goals and will be assisted to problem solve and develop strategies to facilitate their successful implementation. Following each phone call, participants will be provided with a summary of the call and the agreed goals. They will also be provided with relevant resources such as helpful apps or webpages, recipe ideas, and physical activity challenges. The summaries and resources will be available to participants via the LiveSmart user app.

### Follow-up assessment

The follow-up assessment will include the online health questionnaire and a blood sample at the end of the 12-week intervention.

### Outcomes

1. *Change in self-report health behaviours to include:*
  Weight/BMI
  Dietary intake
  Physical activity levels
  Smoking status
  Alcohol consumption
  Supplementation with folic acid 400 μg/5 mg, vitamin D 10 μg
2. *Change in biomarkers of metabolic health to include:*
  Lipid profile
  HbA1c
  Nutritional biomarkers including vitamin D, folate, vitamin B_12_, ferritin
3. *Feasibility and process evaluation to include:*
  - The efficiency of the online recruitment system.
  - Recruitment and retention:
    1. Proportion of women who are eligible to take part in the intervention.
    2. Time to recruit 55 women.
    3. Loss to follow-up.
  - Adequacy of 3-month timeframe to facilitate change in health behaviours and metabolic health.
  - Dose delivered and dose received (the proportion of intended intervention received).
  - Acceptability (if the intervention including online health assessment, home blood test kit, health coaching, intervention materials were well received by participants). Participating women will be asked to complete a digital questionnaire where they rate acceptability of the intervention components on a Likert scale.

### Analysis

Retention will be estimated from recruitment rate, consent rate, withdrawal, and loss to follow-up. For summary statistics, binary and categorical variables will be presented using counts and percentages. The distribution of continuous variables will be assessed using coefficients of skewness and then summarised by mean and standard deviation (SD) or median and interquartile range (IQR) where appropriate. To assess the change in health behaviours (dietary intake, physical activity, weight, smoking, alcohol) and biomarkers of metabolic health, linear and logistic regression will be utilised, with appropriate adjustment. To maintain sample size and power, we will attempt to follow-up all participants; however, if there is missing data present, we will assess the type of missing data and perform the appropriate sensitivity analyses. The data analysis will be performed in Stata (StataCorp LP, College Station, Texas) 16.0.

### Data management and confidentiality

Types of data collected in this study will include participant information on demography, medical and family history, health behaviours including dietary intake and micronutrient supplementation, physical activity, smoking, alcohol use, and metabolic biomarkers. All data will be de-identified in the research dataset. All anonymised data will be stored on a password protected computer. No identifiable data will be included in the final publication. All records will be kept in line with applicable national laws and regulations.

### Ethics

This study has been approved by King’s College London. Study Reference: HR-19/20-13583.

## Discussion

The health of the mother before conception can profoundly influence the risk of complications in pregnancy and may adversely affect the lifelong health of the child [17]. We have found in a large-scale survey that UK women are not adequately prepared for pregnancy. There is no easy way to identify women planning pregnancy and few NHS services to support positive lifestyle behaviour modifications despite enormous demand for advice by this demographic. In addition, targeting women with modifiable risk factors before pregnancy may be a more efficient and beneficial approach.

This study will determine whether it is achievable to link digital tools to identify high risk women planning a pregnancy with a preconception health optimisation programme. Examining change in health behaviours and biomarkers of metabolic health pre- and post-a personalised intervention will determine whether this strategy can improve health in high risk women in the preconception period.

## Data Availability

Not applicable.

## Abbreviations

UK: United Kingdom
NHS: National Health Service
LMUP: London Measure of Unplanned Pregnancy
BMI: Body mass index
GDM: Gestational diabetes mellitus

## References

[1] Stephenson J, Heslehurst N, Hall J, Schoenaker DAJM, Hutchinson J, Cade JE (2018). Before the beginning: nutrition and lifestyle in the preconception period and its importance for future health. Lancet..

[2] Public Health England Fingertips (2018). Public Health Outcomes Framework.

[3] Poston L, Caleyachetty R, Cnattingius S, Corvalán C, Uauy R, Herring S (2016). Preconceptional and maternal obesity: epidemiology and health consequences. Lancet Diabetes Endocrinol.

[4] Morgan KL, Rahman MA, Macey S, Atkinson MD, Hill RA, Khanom A (2014). Obesity in pregnancy: a retrospective prevalence-based study on health service utilisation and costs on the NHS. BMJ Open.

[5] Hanson MA, Bardsley A, De-Regil LM, Moore SE, Oken E, Poston L (2015). The International Federation of Gynecology and Obstetrics (FIGO) Recommendations on Adolescent, Preconception, and Maternal Nutrition: “Think Nutrition First”. Int J Gynecol Obstet.

[6] De-Regil LM, Peña-Rosas JP, Fernández-Gaxiola AC, Rayco-Solon P (2015). Effects and safety of periconceptional oral folate supplementation for preventing birth defects. Cochrane Database Syst Rev.

[7] McCowan LME, Dekker GA, Chan E, Stewart A, Chappell LC, Hunter M, et al. Spontaneous preterm birth and small for gestational age infants in women who stop smoking early in pregnancy: prospective cohort study. BMJ. 2009;338:b1558. 10.1136/bmj.b1081. 10.1136/bmj.b1081 PMC2661373 19325177

[8] Rocca CH, Krishnan S, Barrett G, Wilson M (2010). Measuring pregnancy planning: an assessment of the London Measure of Unplanned Pregnancy among urban, south Indian women. Demogr Res.

[9] Morof D, Steinauer J, Haider S, Liu S, Darney P, Barrett G (2012). Evaluation of the London Measure of Unplanned Pregnancy in a United States population of women. PLoS One.

[10] Hall J, Barrett G, Mbwana N, Copas A, Malata A, Stephenson J. Understanding pregnancy planning in a low-income country setting: validation of the London measure of unplanned pregnancy in Malawi. BMC Pregnancy Childbirth. 2013;13(1). 10.1186/1471-2393-13-200. 10.1186/1471-2393-13-200 PMC4228283 24188251

[11] Public Health England (2018). Making the case for preconception care.

[12] M’hamdi HI, van Voorst SF, Pinxten W, Hilhorst MT, Steegers EAP (2017). Barriers in the uptake and delivery of preconception care: exploring the views of care providers. Matern Child Health J.

[13] Barker M, Dombrowski SU, Colbourn T, Fall CHD, Kriznik NM, Lawrence WT (2018). Intervention strategies to improve nutrition and health behaviours before conception. Lancet..

[14] Chan A-W, Tetzlaff JM, Altman DG, Laupacis A, Gøtzsche PC, Krleža-Jerić K (2013). SPIRIT 2013 statement: defining standard protocol items for clinical trials. Ann Intern Med.

[15] National Institute for Health Research (NIHR). Feasibility and pilot studies, in NIHR NETSCC glossary. https://www.rds-london.nihr.ac.uk/resources/justify-sample-size-for-a-feasibility-study/.

[16] National Insitute for Health and Care Excellence (NICE) (2015). Diabetes in pregnancy: management from preconception to the postnatal period (NG3).

[17] Mikkelsen B, Williams J, Rakovac I, Wickramasinghe K, Hennis A, Shin H-R, et al. Life course approach to prevention and control of non-communicable diseases. BMJ. 2019;364:l257. 10.1136/bmj.l257 PMC6349133 30692103

